# Trabeculectomy with versus without releasable sutures for glaucoma: a meta-analysis of randomized controlled trials

**DOI:** 10.1186/1471-2415-14-41

**Published:** 2014-03-31

**Authors:** Minwen Zhou, Wei Wang, Wenbin Huang, Xiulan Zhang

**Affiliations:** 1 Zhongshan Ophthalmic Center, State Key Laboratory of Ophthalmology, Sun Yat-Sen University, 54S.Xianlie Road, Guangzhou 510060, People’s Republic of China

**Keywords:** Releasable sutures, Trabeculectomy, Meta-analysis

## Abstract

**Background:**

The aim of this study was to compare the efficacy and tolerability of trabeculectomies performed with and without releasable sutures in the treatment of patients with uncontrolled glaucoma.

**Methods:**

A comprehensive literature meta-analysis was performed, comparing trabeculectomies performed with and without releasable sutures. The primary efficacy measure was the weighted mean difference (WMD) in percentage intraocular pressure reduction (IOPR%) at the follow-up end point. The secondary efficacy measure was the risk ratio (*RR*) for complete and qualified success rates of trabeculectomy at the follow-up end point. Trabeculectomy tolerability estimates were measured by the *RR* for adverse events. All the outcomes were reported with a 95% confidence interval (CI).

**Results:**

The WMD of the IOPR% from baseline was −4.56 (range −9.24–0.12) when trabeculectomies without releasable sutures were compared with trabeculectomies with releasable sutures. Trabeculectomies with releasable sutures were associated with numerically greater, but nonsignificant, efficacy in terms of lowered IOP compared with trabeculectomies without releasable sutures. The complete and qualified success rate of the two surgical procedures were comparable, with *RRs* of 0.92 (range 0.80–1.04) and 0.99 (range 0.89–1.11), respectively, at the follow-up endpoints. Trabeculectomies without releasable sutures were associated with a significantly higher frequency of hypotony and flat anterior chambers than trabeculectomies with releasable sutures, with pooled *RRs* of 4.04 (range 1.88–8.68) and 2.57 (range 1.25–5.30), respectively.

**Conclusion:**

Although the two surgical procedures resulted in equivalent efficacy in IOP control, the trabeculectomies performed with releasable sutures were better tolerated than those without releasable sutures.

## Background

Glaucoma is a major eye disease that tends to result in blindness, and trabeculectomy is currently considered an effective surgical procedure to treat uncontrolled glaucoma [1,2]. Although trabeculectomy has been used for the treatment of glaucoma in clinical practice for over 40 years [3], several postoperative complications are associated with this procedure. These include early postoperative excessive filtration, which leads to a shallow or a flat anterior chamber, hypotony, and choroidal detachment [4-6].

Trabeculectomy combined with releasable suture surgery has recently been used to treat uncontrolled glaucoma [7-14]. This technique aims to eliminate or minimize the complications of trabeculectomy without releasable sutures. The use of releasable sutures in trabeculectomy surgery, first mentioned by Schaffer [15], allows the surgeon to close the sclera flap relatively tightly intraoperatively, thereby helping to control the aqueous outflow and to reduce the likelihood of the aforementioned complications. When the postoperative intraocular pressure (IOP) is elevated and decreases after massage, the sutures can be removed to increase the aqueous outflow. This technique is used in clinical practice today. Laser suture lysis can also be used to lower the postoperative IOP, and reduce the likelihood of complications [16]. However, there is a possibility that laser suture lysis, even at a low dose, may cause burns and result in inflammation of the conjunctiva. Laser suture lysis may also lead to conjunctival scars and a flat bleb [17].

Several published randomized controlled trials (RCTs) have compared the efficacy and complications of trabeculectomies performed with and without releasable sutures [8,11,12,14,18,19]. However, these studies included only a modest or a small sample size, and their results were inconclusive. Therefore, to assess the efficacy and complications of these two surgical procedures for the management of uncontrolled glaucoma, we undertook a meta-analysis of all available RCTs.

## Methods

This meta-analysis was performed according to a predetermined protocol, which is described below. Additionally, standard systematic review guidelines, as outlined by the Cochrane Handbook for Systematic Reviews of Interventions [20], were followed at all stages of the process.

### Literature search

Four electronic databases (PubMed, ISI Web of Science, EMBASE, and the Cochrane Library) were searched systematically for studies published before September 1, 2013. The structured search strategies used the following search terms: “glaucoma” or “trabeculectomy” or “filtration surgery” or “filtering surgery” or “filtration operation” or “filtering operation” AND “suture”. The Internet was searched using the Google search engine. A manual search was performed by checking the reference lists of the original reports and the review articles retrieved through the electronic searches to identify studies not yet included in the computerized databases. The final search was carried out on September 1, 2013, without restrictions regarding publication year or language.

### Inclusion and exclusion criteria

The articles were considered eligible if the studies met the following inclusion criteria: (i) study type: RCT; (ii) population: glaucoma patients (but not including secondary glaucoma, congenital glaucoma, or previous intraocular surgery) who failed to respond to conservative therapy; (iii) intervention: trabeculectomies performed with releasable sutures versus trabeculectomy without releasable sutures, with or without the use of antimetabolites; (iv) outcome variables: at least one of the outcomes of interest was included; and (v) follow-up time: ≥6 months. Abstracts from conferences, full texts without raw data available for retrieval, duplicate publications, letters, and reviews were excluded. For publications reporting on the same study population, the article reporting the results of the last endpoint was included, and data that could not be obtained from one publication were obtained from other publications.

### Outcome measures

For efficacy, the primary outcome was the percentage of the IOP reduction (IOPR%). When authors reported the mean and standard deviation (SD) of the IOPR%, we used these values directly. For studies that only reported absolute values for the IOP at baseline and at the endpoint, the IOPR and the SD of the IOPR (SD_IOPR_) were calculated as follows: IOPR = IOP_baseline_ − IOP_endpoint_, SD_IOPR_ = (SD^2^_baseline_ + SD^2^_endpoint_ − SD_baseline_ × SD_endpoint_)^1/2^. The IOPR% and the SD of the IOPR% (SD_IOPR%_) were estimated by IOPR% = IOPR/IOP_baseline_ and SD_IOPR%_ = SD_IOPR_/IOP_baseline_.

The secondary outcome measure was the proportion of patients with complete success, which was defined as the target endpoint IOP without antiglaucoma medication, and with qualified success, which was defined as the target endpoint IOP with or without antiglaucoma medications. We assessed tolerability by considering the proportions of patients with postoperative complications, including hypotony, choroidal effusion, flat anterior chambers, hyphema, and cataracts.

### Data extraction

The data were extracted separately by two reviewers (Z.M.W. and W.W.), and they were rechecked after the first extraction. Discrepancies between the two independent data extractions were resolved by discussion. The information extracted from each study included the authors of the study, year of publication, study design, location of the trial, duration of the study, and number of subjects including their age and sex, their use of antimetabolites, their IOP measurements, and their success rates. The number of withdrawals and patients reporting adverse events were also recorded.

### Risk of bias assessment

Two reviewers (Z.M.W. and W.W.) independently assessed the risk of bias in each trial, following the domain-based evaluation as described in the Cochrane Handbook for Systematic Reviews of Interventions 5.1.0 [20]. Two of the authors subjectively reviewed all the studies and assigned a value of “high”, “low”, or “unclear” to the following factors: (a) selection bias (Was there adequate generation of the randomization sequence? Was allocation concealment satisfactory?); (b) performance and detection bias (Was there blinding of participants, personnel, and outcome assessors?); (c) attrition bias (Were incomplete outcome data sufficiently assessed and dealt with?); (d) reporting bias (Was there evidence of selective outcome reporting?); and (e) other sources of bias (Were any other sources of bias identified?). Any discrepancies between the two authors were discussed, and a consensus was reached.

### Statistical analysis

The outcome measure was assessed on an intent-to-treat (ITT) basis. Given that some of the trials did not report all the outcomes of interest, we conducted a separate meta-analysis for each comparison and outcome. Considering the differences in clinical characteristics among the study groups and the variations in sample size, it was assumed that heterogeneity was present even when no statistical significance was identified. Thus, the data were combined using a random effects model. The weighted mean difference (WMD) of continuous variables and the risk ratio (*RR*) of dichotomous variables were measured. Both outcomes were reported with a 95% confidence interval (CI). Statistical heterogeneity among the studies was evaluated with *χ*^*2*^ and *I*^*2*^ tests. A subgroup analysis was carried out to evaluate the impact of the surgical characteristics on the results. To detect publication biases, we explored asymmetry in funnel plots. These were examined visually. Furthermore, the Begg and Egger measures were calculated [21,22]. *P* < 0.05 was considered statistically significant in the test for an overall effect. The analysis was conducted using the Stata software package (Version 11.0; Stata Corp., College Station, TX).

## Results

### Literature search

**Figure 1:**
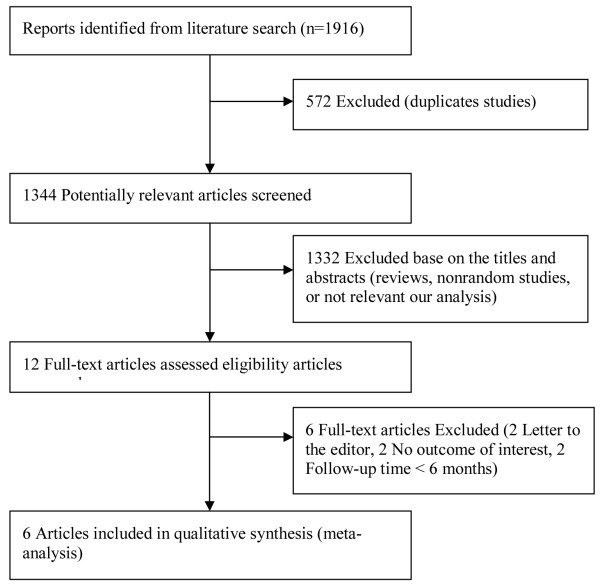
Flow diagram of studies included in this meta-analysis.

A total of 1,916 articles were initially identified. The abstracts were reviewed, and 19 articles with potentially relevant trials were reviewed in their entirety. Subsequently, six articles with full texts that met the inclusion criteria were assessed [8,11,12,14,18,19]. Finally, six studies published between 1998 and 2012 were included in the meta-analysis. Figure 1 provides a flow diagram of the search results.

### Characteristics and baseline of the included studies

The trials were conducted in various countries, including Turkey, Japan, India, and Italy. A total of 296 eyes were included in the meta-analysis: 152 eyes were included in the trabeculectomy without releasable sutures group, and 144 eyes were included in the trabeculectomy with releasable sutures group. The mean age ranged from 53.2 years to 69.6 years. The duration of the studies ranged from 6 months to 14 months. The characteristics of the eligible studies are summarized in Table 1.

**Table 1:** Characteristics of included studies

**Trial (year)**	**Design**	**Country**	Eyes*	Patients*	**Sex (male/female)**		**Age (year)**	**Follow-up (mo)**	**Use of antimetabolite**
					**Without releasable suture**	**Releasable suture**			
Caporossi(2009)	RCT	Italy	33/33	33/33	21/12	17/16	61.7/66.1	14/14	MMC
Kobayashi(2011)	RCT	Japan	25/25	25/25	12/13	14/11	69.2/69.6	12/12	MMC
Simsek (2005)	RCT	Turkey	32/32	32/32	19/13	17/15	61.2/58.3	11.0/11.5	no
Unlu(2000)	RCT	Turkey	20/18	18/17	9/9	9/8	60.9/61.4	8.3/8.1	MMC
Raina(1998)	RCT	India	15/15	15/15	5/10	9/6	54.7/53.2	12/12	no
Aykan(2007)	RCT	Turkey	27/21	24/19	16/11	11/10	59.0/61.0	6/6	MMC

*Trabeculectomy without releasable suture group/trabeculectomy with releasable suture group.

*Abbreviations*: *RCT* prospective randomized controlled trial, *MMC* mitomycin C.

### Risk of bias

**Figure 2:**
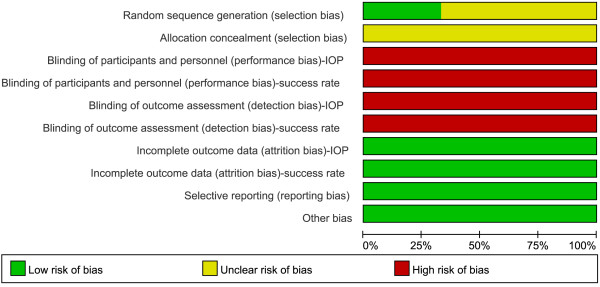
**Risk-of-bias analysis.** Risk-of-bias graph: the authors’ judgments about each risk-of-bias item, presented as percentages across all included studies.

**Figure 3:**
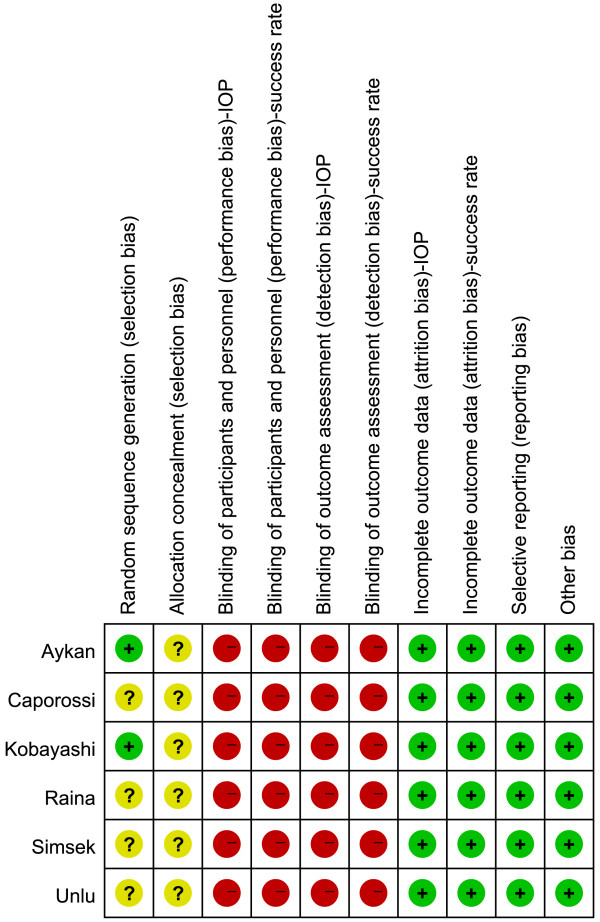
**Risk-of-bias analysis.** Risk-of-bias summary: the authors’ judgments about each risk-of-bias item for the included studies. + low risk; - high risk; ? unclear risk.

The risk-of-bias analysis (Figures 2 and 3) revealed that only two of the included studies [18,19] adequately reported the randomization protocol and that none of the studies described allocation concealment. Additionally, none of the trials sufficiently described their blinding methods. No selective reporting was evident, as it was clear from the published articles that all the main prespecified outcomes were reported. The majority of the trials had a low risk of bias in the “other sources of bias” domain.

### Efficacy analysis

**Figure 4:**
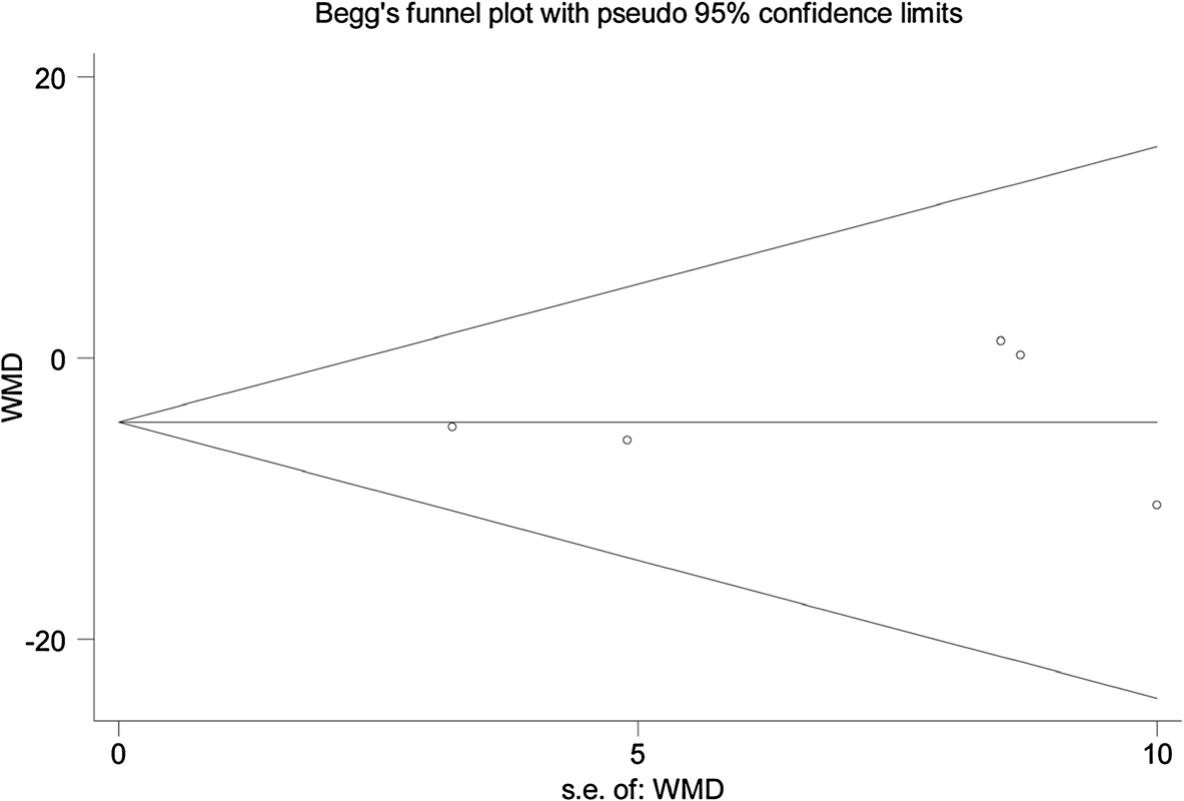
Tests for publication bias for WMD of the percentage of IOP reduction (IOPR%).

The IOPR% of trabeculectomies performed with and without releasable sutures was assessed in the six studies involving 296 eyes. The test for heterogeneity between the studies was insignificant (*χ*^*2*^ = 1.19; *P* = 0.879, *I*^*2*^ = 0.0%). The combined results showed that both surgical procedures significantly decreased the IOP. The IOPR% of the trabeculectomies with releasable sutures were numerically greater compared with the baseline. However, the differences in the IOPR% were not all statistically significant (WMD = −4.56 [−9.24, 0.12]; Table 2). In the subgroup analysis of the use of antimetabolites, the difference between the groups was not statistically significant (Table 3), and there was no statistical heterogeneity between the studies. The funnel plot is shown in Figure 4. All study outcomes were within the 95% CI, and they were distributed symmetrically, showing no evidence of publication bias. Moreover, neither Begg’s test (*P* = 0.806) nor Egger’s test (*P* = 0.661) suggested publication bias in these trials.

**Table 2:** Comparison of percentage IOP reduction from baseline between trabeculectomies without releasable suture and trabeculectomies with releasable suture

**Trial**	**Without releasable suture**		**Releasable suture**		**WMD(random)(95% CI)**
	**No. eyes**	**IOPR (%) [Mean(SD)]**	**No. Eyes**	**IOPR (%) [Mean(SD)]**	
Caporossi (2009)	33	30.74 (18.88)	33	36.59 (20.89)	-5.85 (-15.46, 3.76)
Kobayashi (2011)	25	49.08 (11.43)	25	53.96 (11.29)	-4.88 (-11.18, 1.42)
Unlu (2000)	20	58.20 (23.00)	18	57.98 (29.71)	0.22 (-16.81, 17.25)
Raina (1998)	15	36.90 (23.25)	15	47.35 (30.96)	-10.45 (-30.04, 9.14)
Aykan (2007)	27	57.69 (33.01)	21	56.47 (25.87)	1.22 (-15.44, 17.88)
Total	120		112		-4.56 (-9.24, 0.12)

Test for heterogeneity *χ*^*2*^ = 1.19, *df* = 4, *P* = 0.879.

Test for overall effect *z* = 1.91, *P* = 0.056.

*Abbreviations*: *IOP* intraocular pressure, *CI* confidence interval, *IOPR%* percentage intraocular pressure reduction, *WMD* weighted mean difference.

**Table 3:** Subgroup analyses of percentage reduction in IOP

**Subgroup**	**Studies (n)**	**WMD (Fixed) (95% CI)**	**Heterogeneity**			**Overall effect**	
			**Q**	***P***	**I**^**2**^ **(%)**	**Z**	***P***
All trials	5	-4.56 (-9.24, 0.12)	1.19	0.879	0.00%	1.91	0.056
Use of antimetabolite							
Yes	4	-4.21 (-9.02, 0.61)	0.82	0.844	0.00%	1.71	0.087
No	1	-4.93 (-9.61, -0.26)	0.00	-	-	1.70	0.090

*Abbreviations*: *IOP* intraocular pressure, *WMD* weighted mean difference, *CI* confidence interval.

Of the five studies that reported the probability of complete success, no significant difference was found between the two groups (pooled *RR* = 0.92 [0.80, 1.05]; Table 4). The assessment of publication bias using Begg’s test (*P* = 0.806) and Egger’s test (*P* = 0.822) showed that no publication bias existed among the included trials. Additionally, the difference in the qualified success rate between the two groups was not statistically significant (pooled *RR* = 0.99 [0.89, 1.11]; Table 4). Publication bias in this outcome was not assessed because of the limited number of studies included.

**Table 4:** Comparison of complete success and qualified success between trabeculectomies without releasable suture and trabeculectomies with releasable suture

**Trial**	**Studies ( *n *)**	**Success rate, n/N(%)**		**RR (95% CI)**	**Heterogeneity**			**Overall effect**	
		**Without releasable suture**	**Releasable suture**		**Q**	***P***	**I**^**2**^ **(%)**	**Z**	***P***
Complete success									
All trials	5	90/119 (75.63%)	92/111 (82.88%)	0.92 (0.80,1.04)	1.31	0.860	0.00%	1.31	0.191
Qualified success									
All trials	4	83/87 (95.40%)	76/79 (96.20%)	0.99 (0.89,1.11)	0.34	0.560	0.00%	0.20	0.845

*Abbreviations*: *RR* risk ratio, *CI* confidence interval.

### Tolerability analysis

A comparison of adverse events between the RCTs of the trabeculectomies with and without releasable sutures is presented in Table 5. Hypotony, a flat anterior chamber, choroidal effusion, hyphema, and cataract formation were the commonly reported postoperative complications. Trabeculectomy without releasable sutures was associated with a significantly higher frequency of hypotony and a flat anterior chamber than trabeculectomy with releasable sutures, with pooled *RRs* of 4.04 (range 1.88–8.68) and 2.57 (range 1.25–5.30), respectively. However, no significant differences in the incidence of choroidal effusion, hyphema, and cataract formation were found between the two groups, with pooled ORs of 2.51 (range 0.89–7.07), 0.93 (range 0.43–2.03), and 1.59 (range 0.48–5.30), respectively.

**Table 5:** Comparison of adverse events between trabeculectomies without releasable suture and trabeculectomies with releasable suture

**Adverse events**	**Studies ( *n *)**	**Crude event rate,** ***n*/N**		**RR (95% CI)**	**Heterogeneity**			**Overall effect**	
		**Without releasable suture**	**Releasable suture**		**Q**	***P***	**I**^**2**^ **(%)**	**Z**	***P***
Hypotony	3	28/72	6/72	4.04 (1.88,8.68)	1.02	0.602	0.00%	3.58	<0.001
Flat anterior chamber	5	37/137	12/129	2.57 (1.25,5.30)	5.35	0.254	25.20%	2.56	0.010
Choroidal effusion	4	10/93	3/91	2.51 (0.89,7.07)	0.04	0.998	0.00%	1.74	0.082
Hyphema	6	12/152	11/144	0.93 (0.43,2.03)	3.95	0.557	0.00%	0.18	0.855
Cataract formation	3	7/62	4/54	1.59 (0.48,5.30)	0.58	0.750	0.00%	0.75	0.451

*Abbreviations*: *RR* risk ratio, *CI* confidence interval.

## Discussion

Trabeculectomy is still considered the mainstay for medically uncontrolled glaucoma [23]. Several modifications and variations have been developed to maximize the benefits of treatment while minimizing adverse events [19,23,24]. However, this technique is associated with complications. Trabeculectomy supplemented with releasable sutures can decrease the IOP rate and reduce post-trabeculectomy complications during the early postoperative period [14].

Previous studies have prospectively evaluated the efficacy and safety of trabeculectomies performed with and without releasable sutures [8,11,12,14,18,19]. All the trials showed that the techniques were comparably efficacious in lowering the IOP. However, the various trials showed different incidence rates of complications, and some trials included only small sample sizes, which hindered the ability to draw conclusions for clinical practice. Therefore, the present meta-analysis was undertaken to assess the efficacy and tolerability of both surgical procedures in the treatment of uncontrolled glaucoma.

To the best of our knowledge, this is the first meta-analysis to explore the efficacy and tolerability of trabeculectomy performed with the releasable suture procedure. We reviewed six RCTs of trabeculectomies, with and without releasable sutures in patients with uncontrolled glaucoma. The pooled results from the meta-analysis, using a random effects model, suggested that an intraoperative releasable suture application is comparable to trabeculectomies without releasable sutures in lowering IOP and that it is comparable to trabeculectomies without releasable sutures in the complete and qualified success rates. Furthermore, the results of the subgroup analyses were quite similar and robust.

This releasable suture surgical procedure was developed as an alternative to trabeculectomy without releasable sutures, mainly to overcome possible complications, such as hypotony, a shallow, flat anterior chamber, and choroidal detachment. We found that the releasable suture surgical procedure reduced these complications when compared with the traditional practice, with a statistically significant difference in the incidence of hypotony and flat anterior chambers between the two groups. Inadequate tension in the scleral sutures was responsible for the majority of cases with flat anterior chambers and hypotony in an otherwise uneventful trabeculectomy [25]. The lower incidence of complications in the group with releasable sutures was mainly due to the relatively tight sclera flap releasable sutures, which can control the aqueous outflow more easily. The current study provides interesting findings that could be useful in the selection of surgical procedures.

The first strength of the present analysis is that it focused on a direct comparison between trabeculectomies performed with and without releasable sutures, rather than on an indirect comparison. Second, the likelihood of bias was minimized by developing a detailed protocol before initiating the study, by performing a meticulous search for published studies, and by using explicit methods for study selection, data extraction, quality assessment, and statistical analysis. Finally, the subgroup analysis demonstrated that the conclusions of this analysis are robust.

One major limitation of this analysis is that we cannot fully exclude publication bias. To avoid publication bias, we conducted not only an electronic search but also a manual search to identify all potentially relevant articles, including those that were published or nonpublished. Unfortunately, it is possible that we failed to include some papers, especially those published in other languages. The second limitation is that although no significant heterogeneity was found, the studies were carried out with small or very small sample sizes, inadequate allocation concealment, and inadequate or no double-blinding. These factors can affect the interpretation of the results. The third limitation is that the analyses of clinically relevant outcome measures were based on data pooled from trials with different follow-up periods. To decrease this bias, we included only studies with follow-up times of at least 6 months. Another potential source of heterogeneity in the results was the assessment criterion for success. Success was defined as a target endpoint IOP. Although such assessments of success are widely used as outcome measures in clinical trials, further research is still needed to determine their validity, reliability, and sensitivity to ensure that the best is chosen. Finally, with regard to the complication of cataract formation, the included studies seldom mentioned the cataract grading. Cataract grading following trabeculectomies performed with and without releasable sutures should be the focus of future studies.

## Conclusions

In conclusion, the results of this meta-analysis of six RCTs indicated that trabeculectomies with releasable sutures showed equivalent efficacy to trabeculectomies without releasable sutures in controlling the IOP and that the proportions of patients in both groups who achieved the target IOP were comparable. The analysis of the incidence of postoperative complications showed that a trabeculectomy with releasable sutures is safer than a trabeculectomy without releasable sutures.

## Competing interests

The authors declare that they have no competing interests.

## Authors’ contributions

All authors conceived of and designed the experimental protocol. MZ and WW collected the data. All authors were involved in the analysis. MZ wrote the first draft of the manuscript. MZ, WW and XZ reviewed and revised the manuscript and produced the final version. All authors read and approved the final manuscript.

## Pre-publication history

The pre-publication history for this paper can be accessed here:

http://www.biomedcentral.com/1471-2415/14/41/prepub

## References

[B1] Bertrand V Fieuws S Stalmans I Zeyen T Rates of visual field loss before and after trabeculectomy Acta Ophthalmol 2014 92 116 120 10.1111/aos.12073 23551517

[B2] Kirwan JF Lockwood AJ Shah P Macleod A Broadway DC King AJ McNaught AI Agrawal P Trabeculectomy in the 21st century: a multicenter analysis Ophthalmol 2013 120 2532 2539 10.1016/j.ophtha.2013.07.049 24070811

[B3] Back M Trabeculectomy for glaucoma Arch Ophthalmol 1975 93 1372 1200902 10.1001/archopht.1975.01010020994011

[B4] Dreyer EB Post-trabeculectomy hypotony Ophthalmol 1997 104 1367 10.1016/s0161-6420(97)30133-x 9307624

[B5] Alemu B Trabeculectomy: complications and success in IOP control Ethiop Med J 1997 35 1 11 9293142

[B6] Berke SJ Bellows AR Shingleton BJ Richter CU Hutchinson BT Chronic and recurrent choroidal detachment after glaucoma filtering surgery Ophthalmol 1987 94 154 162 10.1016/s0161-6420(87)33482-7 3574881

[B7] Prevezas DC Melissurgos ID Adjustable temporal suture in trabeculectomy with scleral flap Ophthalmic Surg Lasers 1997 28 745 750 9304637

[B8] Raina UK Tuli D Trabeculectomy with releasable sutures: a prospective, randomized pilot study Arch Ophthalmol 1998 116 1288 1293 10.1001/archopht.116.10.1288 9790625

[B9] Kolker AE Kass MA Rait JL Trabeculectomy with releasable sutures Arch Ophthalmol 1994 112 62 66 10.1001/archopht.1994.01090130072020 8285895

[B10] Matlach J Hoffmann N Freiberg FJ Grehn F Klink T Comparative study of trabeculectomy using single sutures versus releasable sutures Clin Ophthalmol 2012 6 1019 1027 22848142 10.2147/OPTH.S32503 PMC3402124

[B11] Caporossi A Balestrazzi A Malandrini A Tosi GM Caporossi T Frezzotti P Lomurno L A randomized prospective study comparing trabeculectomy with and without the use of a new removable suture Int Ophthalmol 2009 29 359 365 10.1007/s10792-008-9245-z 18553060

[B12] Simsek T Citirik M Batman A Mutevelli S Zilelioglu O Efficacy and complications of releasable suture trabeculectomy and standard trabeculectomy Int Ophthalmol 2005 26 9 14 16779569 10.1007/s10792-006-0002-x

[B13] Liang YB Feng MY Meng HL Fan SJ Wang X Xie LL Yi P Tang X Wang NL Thomas R Early efficacy and complications of releasable sutures for trabeculectomy in primary angle-closure glaucoma: a randomized clinical trial J Glaucoma 2014 23 136 141 10.1097/IJG.0b013e31826981c9 23059481

[B14] Unlu K Aksunger A Soker S Ertem M Mitomycin C primary trabeculectomy with releasable sutures in primary glaucoma Jpn J Ophthalmol 2000 44 524 529 10.1016/S0021-5155(00)00221-5 11033132

[B15] Shaffer RN Hetherington JJ Hoskins HJ Guarded thermal sclerostomy Am J Ophthalmol 1971 72 769 772 4938988 10.1016/0002-9394(71)90016-x

[B16] Savage JA Condon GP Lytle RA Simmons RJ Laser suture lysis after trabeculectomy Ophthalmol 1988 95 1631 1638 10.1016/S0161-6420(88)32964-7 3068601

[B17] L’Esperance FJ The ocular histopathologic effect of krypton and argon laser radiation Am J Ophthalmol 1969 68 263 273 5798807 10.1016/0002-9394(69)94069-0

[B18] Kobayashi H Kobayashi K A comparison of the intraocular pressure lowering effect of adjustable suture versus laser suture lysis for trabeculectomy J Glaucoma 2011 20 228 233 10.1097/IJG.0b013e3181e3d0e4 20577111

[B19] Aykan U Bilge AH Akin T Certel I Bayer A Laser suture lysis or releasable sutures after trabeculectomy J Glaucoma 2007 16 240 245 10.1097/IJG.0b013e31802d6ded 17473738

[B20] Higgins J Green S Cochrane Handbook for Systematic Reviews of Interventions Version 5.1.0.: The Cochrane Collaboration 2011 Available: http://www.cochrane-handbook.org

[B21] Egger M Davey SG Schneider M Minder C Bias in meta-analysis detected by a simple, graphical test BMJ 1997 315 629 634 10.1136/bmj.315.7109.629 9310563 PMC2127453

[B22] Begg CB Mazumdar M Operating characteristics of a rank correlation test for publication bias Biometrics 1994 50 1088 1101 10.2307/2533446 7786990

[B23] Wells AP Bunce C Khaw PT Flap and suture manipulation after trabeculectomy with adjustable sutures: titration of flow and intraocular pressure in guarded filtration surgery J Glaucoma 2004 13 400 406 10.1097/01.ijg.0000133387.82126.7c 15354079

[B24] Jones E Clarke J Khaw PT Recent advances in trabeculectomy technique Curr Opin Ophthalmol 2005 16 107 113 10.1097/01.icu.0000156138.05323.6f 15744141

[B25] Watkins PJ Brubaker RF Comparison of partial-thickness and full-thickness filtration procedures in open-angle glaucoma Am J Ophthalmol 1978 86 756 761 736072 10.1016/0002-9394(78)90117-4

